# Anaemia among adolescents: assessing a public health concern in Lao PDR

**DOI:** 10.1080/16549716.2020.1786997

**Published:** 2020-08-03

**Authors:** Sengchanh Kounnavong, Manithong Vonglokham, Thidatheb Kounnavong, Djan Daniel Kwadwo, Dirk R. Essink

**Affiliations:** a Lao Tropical and Public Health institute, Ministry of Health, Lao People's Democratic Republic; b Global Health Department, Nagasaki University, Nagasaki, Japan; c Athena Institute for Research on Innovation and Communication in Health and Life Sciences, Faculty Sciences, Vrije University Amsterdam, Amsterdam, Netherlands

**Keywords:** LEARN: Sexual Reproductive Health, ANC and Nutrition, Adolescence, anaemia, public health, sex differences, schools

## Abstract

**Background:**

Lao PDR has identified the need to target adolescent public health concerns. Adolescents suffering from poor health and nutrition during rapid growth and development may be at risk of anaemia due to high iron requirements and the rapid depletion of body iron stores.

**Objective:**

This study assessed the prevalence and severity of anaemia among school adolescents in Pholhong district, Vientiane province, Lao PDR.

**Methods:**

A school-based cross-sectional study was conducted among 405 randomly selected school adolescents across 8 high schools in a rural district of Vientiane province. Adolescents aged 10–18, both male and female, were recruited. Haemoglobin concentration from capillary blood was measured. Descriptive statistics were computed for prevalence of anaemia, anthropometric measurements, socio-economic and socio-demographic variables. Multivariate logistic regression analysis was performed to identify determinants of anaemia among subjects. Results were expressed as odds ratios and 95% confidence intervals.

**Results:**

The prevalence of anaemia among adolescents in the study area was 19.4%. There was no difference in the prevalence of anaemia between younger and older adolescents, but the prevalence of anaemia was higher in female adolescents than among males (crude OR = 3.91, 95% CI 2.20 to 6.96). On univariate analysis, coming from an ethnic minority household was found to be significantly associated with anaemia among these adolescents (p < 0.05). After adjusting for other variables, only the effect of sex remained significant. Other factors showing no significant association with anaemia included parents’ employment status, family size, and living conditions.

**Conclusions:**

The prevalence of anaemia in this population is of public health concern with adolescents of both sexes at risk of developing anaemia. The national nutrition programme to control and manage anaemia by distributing a weekly iron and folate supplement for adolescent girls together with a deworming programme twice per year appears to have partly successful but could be strengthened.

## Background

The Sustainable Development Goals pay specific attention to adolescents – those aged 10–19 years [1,2]. Adolescent-specific targets relate to health and wellbeing, nutrition and food security, and gender equality [3]. As a transition period between childhood and adulthood, adolescence is characterized by rapid growth and development [4] that affects people also in the future years of their life cycle [5].

Anaemia is a public health concern [6] because of its adverse effects on cognitive performance, physical capacity and work performance [7]. Anaemia is more common in adolescents, particularly those living in developing countries [8]. Among adolescents, anaemia affects not only the present health status but can also result in deleterious effects appearing later in life [9].

The combination of rapid growth, menstrual blood loss, and often inadequate dietary intake of iron places adolescent females at particular risk of iron deficiencies [10]. Iron deficiency anaemia (IDA), especially when severe, is associated with increased risk of preterm labour, low birthweight, and child and maternal mortality, and it may predispose adolescents to infection and heart failure [11].

Anaemia affects half a billion women of reproductive age worldwide. In 2011, 29% (496 million) of non-pregnant women and 38% (32.4 million) of pregnant women aged 15–49 years were anaemic [9]. The highest prevalence of anaemia is found in South Asia and in Central and West Africa. Estimates in high-risk populations suggest that the total anaemia prevalence may be as high as 50% to 80%, with as many as 10% to 20% having moderate to severe anaemia. The prevalence of anaemia is consistently higher in people with low socioeconomic status, low body weight, and in females who have recently given birth.

Reports show that globally, the prevalence of anaemia fell by 12% between 1995 and 2011 – from 33% to 29% in non-pregnant women and from 43% to 38% in pregnant women, indicating that progress is possible but presently insufficient to meet the goals. All countries are expected to review national policies, infrastructure and resources and then to act to implement strategies for the prevention and control of anaemia [12].

Lao PDR has been classified as Least Developed Country [13], and has entered the ASEAN Economic Community, which has resulted in identification of the need to target investments in young people (10–24 years old). Young people comprise 31.9% of the population, with 66.4% of them living in rural areas [14]. There is a need to focus on nutrition from a life-cycle perspective. Lao PDR also has issues of teenage pregnancy and early marriage, which leads to adverse nutritional outcomes in the children and young mothers. These problems need to be addressed. Current research often ignores the high numbers of adolescent girls among pregnant women, and the increased nutritional needs of adolescents, especially of girls. Again, little research has focused on malnutrition and anaemia among adolescents. Reliable information about nutritional status is essential to identify potentially critical nutrients and the population groups at risk of deﬁciency, as a basis to develop effective public health policies and plans to avert nutrition patterns that may result in morbidity and mortality.

A food-based approach has been defined as one of the most effective programmes to combat or reduce the prevalence of anaemia. This paper describes a study to identify the nutrition problems in the community, in particular, to assess the nutritional and haemoglobin status among adolescent schoolgirls to develop food-based recommendations (FBRs) in rural Pholhong district, Vientiane province, Lao PDR.

## Conceptual framework

This study focuses on factors which might be associated with anaemia among adolescents, including socio-cultural and economic demographic conditions, body mass index, infectious and parasitic diseases [15–17].

## Methods

### Study area and period

**Figure 1.**
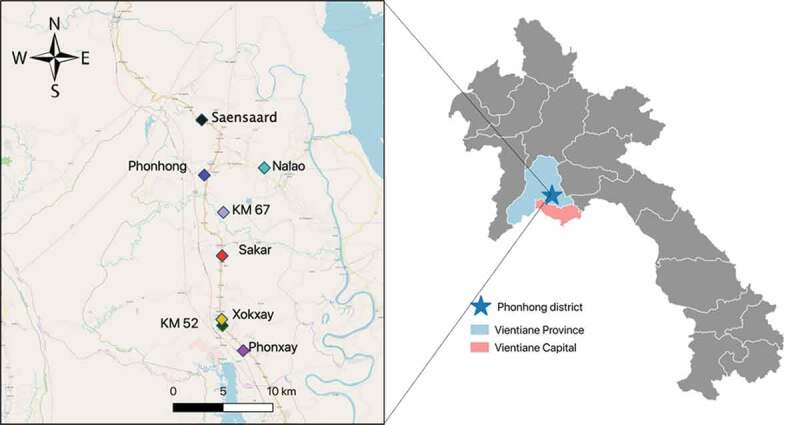
Map of Lao PDR and study area.

A school-based descriptive cross-sectional study was conducted from March to April 2019 in the Lao Tropical and Public Health Institute Nutrition Project areas of Vientiane province, Lao PDR. Pholhong district is one of the 11 districts in Vientiane province, located 60 km north of the capital city Vientiane as shown in Figure 1; it has a total population of 65,200, 37 high schools, and 11 markets [14]. Pholhong district was purposively selected as study site to perform Food-Based Recommendations piloting as it is a place where food is available and more accessible compared to other districts in the province.

### Sampling

The sample size was calculated as a single population with the following assumptions: 42.6% prevalence of anaemia among adolescent girls 15–19 years [18], a confidence interval of 95%, and desired precision (d) of 5%, giving a sample size of 376. Assuming a non-response rate of 10% based on previous experience, the overall sample size planned for the study was 414.

The district has 37 high schools, of which 8 schools were selected for the study, using a simple random sampling method. There were 9,293 adolescents attending 37 high schools in the 2018/2019 academic year. A proportional allocation method was used for determining the number of adolescent students to include from each school. The selection of each study subject (student) at each school was conducted by table of random numbers based on the list of students available in the school registry, after excluding non-adolescent students. Participation in the study was voluntary. Written informed consent was obtained from all 405 apparently healthy individuals, who could communicate in the Lao language and, for the girls, had already had their menarche (first menstruation) and were not pregnant.

### Data collection materials

To avoid non-response bias, recall bias, and interviewer bias, the structured questionnaire was pre-tested in one school not included in the study. Data collectors, who performed data collection, were trained on interviewing techniques following all procedures for quality assurance. Field supervisors for the study were those with nutrition training of community nutrition. Interviews were done with 405 school adolescents using a structured questionnaire, which captured the respondents’ age, years of education, address, date of birth, parents’ occupation, and information regarding the food and dietary habits. In addition, anthropometric assessment, capillary blood testing for haemoglobin, and stool examination were performed for all study subjects. Their dietary intake was also assessed over one week.

### Measurements

#### Blood test

A portable haemoglobin meter (HemoCue AB, Angelholm, Sweden) was used to determine haemoglobin concentration from a capillary blood sample collected aseptically by sterile single-use disposable lancet from the fingertip. Routine safety measures were taken during blood collection. Anaemia status was determined based on the WHO classification using the haemoglobin level of the respondents. Anaemia was considered as present for all adolescents aged ≤11 years with a haemoglobin value <11.5 g/dL, for all adolescents age 12–14 and female adolescents ≥15 years of age with a haemoglobin value of less than 12 g/dL and for male adolescents > 15 years of age, a haemoglobin value of less than 13 g/dL. The severity of anaemia was also classified based on the WHO standard as severe: Hb level below 7 g/dL; moderate: Hb level 7 g/dL to 9.9 g/dL or mild: Hb level 10 g/dL to 11.4 g/dL in adolescents aged ≤11 years, Hb level 10 g/dL to 11.9 g/dL in all adolescents 12 to14 years of age, and females ≥15 years, and Hb level 10 g/dL to 12.9 g/dL in males ≥15 years [9,10].

#### Anthropometric measurements

Measurements of height and weight were taken according to WHO guidelines. Weights were measured to the nearest 0.1 kg on a battery powered digital scale (SECA, UNICEF, Copenhagen) and height was measured to the nearest 0.1 cm using a wooden height-measuring board with a sliding head bar following standard anthropometric techniques [19]. Anthropometric indicators used in this study were BMI for age z-score (BAZ) and height for age z-score (HAZ). Adolescents below the −2 HAZ score were classified as stunted and those with BAZ score less than −2 were classified as thin.

#### Stool examination

Standard operating procedures (SOPs) and manufacturers’ instructions were strictly followed for all laboratory activities and reagents were checked for expiry dates. To assess helminth infection, containers were distributed to each adolescent and they were asked to collect and bring a sample of their faeces at the time of data collection. The samples were stained and examined within 1 hr of staining by the Kato-Katz method for the presence of Minute Intestinal Flukes (*Opisthorchis viverrini*, OV), geo-helminths (*Trichuris trichuria, Ascaris lumbricoides*, hookworm, and *Strongyloides stercoralis), E. coli* and *Taenia*.

#### Data processing and data analysis

**Figure 2.**
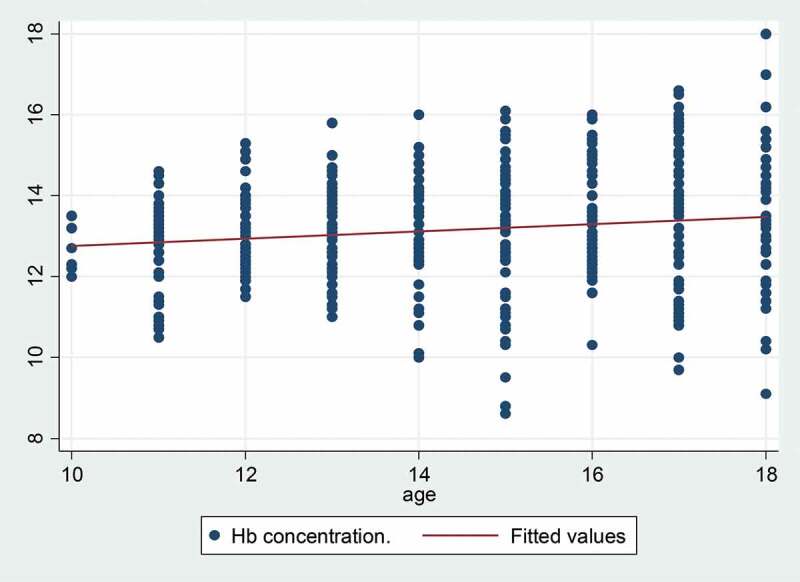
Distribution of haemoglobin level by age of adolescents (in year), (n = 402). Mean: 13.2 g/dL (SD 1.4 g/dL, Max: 18.0 g/dL, Min: 8.6 g/dL); Median: 13.1 g/dL.

For data entry, template formats were prepared in the Census and Survey Processing System (CSPro developed by United Sates Census Bureau) and data were singly entered. Data were coded, examined and prepared for analysis with STATA, version 16. The sociodemographic, health, and nutrition characteristics of the study population were summarized as the mean and SD for continuous variables and as the frequency for categorical variables. The chi-square (χ2) test and Fisher’s exact test were used to compare categorical variables between groups (Figure 2).Bivariate analysis of the association between anaemia and potential risk factors was conducted using logistic regression. Variables associated with anaemia at p < 0.2 were incorporated in multiple regression models. The results were expressed as the odds ratio (OR) and 95% confidence interval (95% CI). The significance level was set at p < 0.05 for all tests.

For this study, the maximum family size recorded was five or more. Adolescents were categorized as younger (10–14 years) and older (15–18 years). The main occupations of adolescents’ fathers and mothers were classified as self-employed (farmer, worker, seller) or officer.

## Results

From a total of 414 students invited to participate in the study, 405 (97.8%) responded to the questionnaire. However, 3 of the 405 refused consent to the blood test, so 402 cases were entered for final analysis. More than half of the participants were female (50.9%), while 206 (51.2%) were in the age range 15–18 years, with a mean (SD) of 14.5 (2.2) years. Table 1 summarizes the socio-demographic and health characteristics of the study subjects in the eight schools. Regarding household characteristics, most (312; 77.6%) of the respondents’ households were ethnic Lao. Most of the students (331; 82.3%) stayed with their parents and nearly one-third (257; 63.9%) were from households with a family size of less than five people. Most of the fathers (72.8%) and mothers (87.4%) were self-employed, working as farmers, labourer, or sellers. Most of the respondents (320; 79.6%) reported that their usual daily meal frequency was three times per day and above. Almost all reported consumption of fruit (381; 94.8%) and vegetables (354; 88.1%) less than four times in the last three days. Only 18.5% of the adolescents always used soap when washing their hands. Overall, the results showed some heterogeneity among the eight schools in terms of sex, age, fathers’ and mothers’ occupation, family size, ethnicity, living status, daily meal frequencies, and vegetable consumption.

**Table 1.** Characteristics of school adolescents in Pholhong district, Vientiane province. April 2019 (n = 405).

		School location (No. of students) and (%)									
Characteristics		Sansaard (n = 40)	Nalao (n = 40)	Pholhong (n = 41)	Km 67 (n = 60)	Sakar (n = 40)	Xokxay (n = 45)	KM52 (n = 79)	Phonxay (n = 60)	Total (n = 405)	p-value
Sex	Male	19 (47.5)	20 (50.0)	20 (48.7)	30 (50.0)	20 (50.0)	20 (44.4)	40 (50.3	30 (50.0)	199 (49.1)	0.999
	Female	21 (52.5)	20 (50.0)	21 (51.2)	30 (50.0)	20 (50.0)	25 (55.5)	39 (49.3)	30 (50.0)	206 (50.9)	
Age	10–14	-	37 (92.5)	20 (48.7)	29 (48.3)	18 (45.0)	26 (57.8)	39 (49.4)	30 (50.0)	199 (49.1)	**0.000**
	15–18	40 (100.0)	3 (7.5)	21 (51.2)	31 (51.7)	22 (55.0)	19 (42.2)	40 (50.6)	30 (50.0)	206 (50.9)	
Mean age	14.5 ± 2.2										
Nutrition status	HAZ>-2	36 (90.0)	35 (87.5)	36 (87.8)	54(90.0)	37 (92.5)	43 (95.6)	62 (78.5)	54 (90.0)	357 (88.2)	0.152
	HAZ<-2	4 (10.0)	5 (12.5)	5 (12.2)	6 (10.0)	3 (7.5)	2 (4.4)	17 (21.5)	6 (10.0)	48 (11.8)	
	BAZ>-2	40 (100.0)	35 (87.5)	41 (100.0)	60 (100.0)	37 (92.5)	44 (97.8)	77 (97.5)	56 (93.3)	390 (96.3)	0.013
	BAZ<-2	0	5 (12.5)	0	0	3 (7.5)	1 (2.2)	2 (2.5)	4 (6.7)	15 (3.7)	
Intestinal	Negative	24 (60.0)	23 (57.5)	24 (58.5)	47 (78.3)	28 (70.0)	28 (62.2)	55 (69.6)	38 (63.3)	267 (65.9)	0.313
parasites	Positive	16 (40.0)	17 (42.5)	17 (41.5)	13 (21.7)	12 (30.0)	17 (37.8)	24 (30.4)	22 (36.7)	138 (34.1)	
Father’s occupation	Self-employed	32 (80.0)	34 (85.0)	34 (82.9)	31 (51.7)	34 (85.0)	30 (66.7)	64 (81.0)	36 (60.0)	295 (72.8)	**0.000**
	Officer	8 (20.0)	6 (15.9)	7 (17.1)	29 (48.3)	6 (15.0)	15 (33.3)	15 (19.0)	24 (40.0)	110 (27.2)	
Mother’s occupation	Self-employed	35 (87.5)	37 (92.5)	32(78.0)	44 (73.3)	39 (97.5)	40 (88.9)	74 (93.7)	53 (88.3)	354 (87.4)	**0.003**
	Officer	5 (12.5)	3 (7.5)	9 (21.9)	16 (26.7)	1 (≤2.5)	5 911.1)	5 (6.3)	7 (11.7)	51 (12.6)	
Family size	<5	32 (80.0)	33 (82.5)	29 (70.7)	41 (68.3)	25 (62.5)	26 (57.8)	31 (39.2)	41 (68.3)	258(63.7)	**0.000**
	≥5	8 (20.0)	7 (17.5)	12 (29.3)	19 (31.7)	15 (37.5)	19 (42.2)	48 (60.8)	19 (31.7)	147 (36.3)	
Ethnicity	Lao	40 (100.0)	40 (100.0)	38 (92.7)	43 (71.7)	36 (90.0)	39 (86.7)	21 (26.6)	57 (95.0)	314 (77.5)	**0.000**
	Minorities	0	0	3 (7.3)	17 (28.3)	4 (10.0)	6 (13.3)	58 (73.4)	3 (5.0)	91 (22.5)	
Daily meal frequency	≤2 times ≥3times	9 (22.5) 31 (77.5)	15 (37.5) 25 (62.5)	8 (20.0) 32 (80.0)	13 (21.7) 47 (78.3)	7 (17.5) 33 (95.5)	2 (4.4) 43 (87.3)	10 (12.6) 69 (87.3)	17 (28.3) 43 (77.7)	81 (20.1) 323 (79.9)	**0.005**
Vegetable	<4	34 (85.0)	40 (100.0)	36 (90.0)	48 (80.0)	36 (90.0)	30 (66.7)	77 (97.5)	56 (93.3)	357 (88.4)	**0.000**
consumption	≥4	6 (15.0)	0	4 (10.0)	12 (20.0)	4 (10.0)	15 (33.3)	2 (2.5)	4 (6.7)	47 (11.6)	
Fruit	<3	40 (100.0)	37 (92.5)	40 (97.6)	54(90.0)	38 (95.0)	43 (95.6)	77 (98.7)	57(96.6)	384 (96.7)	0.631
consumption	≥3	0	3 (7.5)	1 (2.44)	6 (10.0)	2 (5.0)	2 (4.4)	1 (1.3)	2 (3.4)	13 (3.27)	
Handwash With soap	No	30 (75.0)	34 (85.0)	29 (70.7)	51 (85.0)	28 (70.0)	40 (88.9)	67 (84.8)	51 (85.0)	330 (81.5)	0.127
	Yes, always	10 (25.0)	6 (15.0)	12 (29.3)	9 (15.0)	12 (30.0)	5 (11.1)	12 (15.2)	9 (15.0)	75 (18.5)	
Living status	With parent(s)	34 (85.0)	36 (980.0)	35 (85.4)	36 (60.0)	32 (80.0)	35 (77.8)	71 (89.8)	55 (91.7)	334 (82.5)	**0.000**
	With relative(s)	6 (15.0)	4 (10.0)	6 (14.6)	24 (40.0)	8 920)	10 (22.2)	8 (10.1)	5 (8.3)	71 (17.5)	

HAZ – height for age Z score; BAZ – body mass index for age Z score.

Regarding the nutrition status of the school adolescents, 11.8% suffered from stunting and 3.7% from underweight. Among the study participants, 61 (14.9%) had BAZ greater than +2. The results from stool examination demonstrated that 135 (33.6%) study subjects had at least one parasite; the predominant parasite was *Opisthorchis viverrini*, detected in the stool of 121 (89.6%) of the 135 adolescents with parasites. Only 3.6% of them had hookworms and 2.9% of had *Ascaris lumbricoides*.

The mean (SD) haemoglobin concentration of the 402 adolescents was 13.2 (1.4) g/dL, while 19.4% had values below the anaemia cut-off point. Most cases were mildly anaemic and no severely anaemic adolescents were found in this study. Table 2 shows the haemoglobin concentration and prevalence of anaemia based on the subjects’ general sociodemographic, health, and nutrition characteristics. Regarding the association between individual characteristics and anaemia among adolescents, there was no difference in the prevalence of anaemia between younger and older adolescents, but the prevalence of anaemia was higher in female adolescents than among males (crude OR = 3.91, 95% CI (2.20 to 6.96)). Other factors showing no significant association with anaemia were parents’ working status, family size, living conditions, hand-washing with soap, frequency of fruit and vegetable consumption, and daily meal frequency. On univariate analysis, coming from an ethnic minority household was found to be significantly associated with anaemia among these adolescents (p < 0.05). After adjusting for other variables, only the effects of sex remained significant.

**Table 2.** Mean haemoglobin level (SD) and unadjusted and adjusted odds ratios (and 95% CI) for association between anaemia and socio-demographic and nutrition risk factors among adolescents (n = 402) in Pholhong district, Vientiane province, April 2019.

			Anaemia Odds Ratio (95% CI)		
Characteristics		Haemoglobin concentration (g/dL) [mean (SD)]	N (%)	Unadjusted	Adjusted^a^
Sex	Male	13.7 (1.4)	19 (9.6)*	Reference	
	Female	12.6 (1.2)	59 (28.9)	**3.91 (2.20–6.96)**	**3.77 (2.14–6.65)**
Age	10–14	13.0 (1.2)	35 (17.8)	1.19 (0.71–2.00)	
	15–18	13.3 (1.6)	43 (20.8)	Reference	
Height for age Z score	< −2	12.9 (1.6)	13 (27.0)	1.84 (0.88–3.83)	
	≥ −2	13.2 (1.4)	65 (18.3)	Reference	
BMI for age Z score	< −2	12.8 (0.9)	2 (13.3)	0.49 (0.10–2.34)	
	≥ −2	13.2 (1.5)	76 (19.6)	Reference	
Stool parasites**	Yes	13.2 (1.5)	28 (20.7)	1.18 (0.64–2.17)	
	No	13.1 (1.4)	50 (18.7)	Reference	
Mother’s main occupation	Self-employed (Farmer/labour/seller)	13.2 (1.5)	71 (20.2)	Reference	
	Officer	13.0 (1.1)	7 (13.7)	0.57 (0.21–1.55)	
Father’s main occupation	Self-employed (Farmer/labour/seller)	13.2 (1.5)	60 (20.5)	Reference	
	Officer	13.0 (1.1)	18 (16.4)	0.74 (0.36–1.50)	
Ethnicity	Lao	13.0 (1.4)	67 (21.4)	Reference	
	Minorities	13.6 (1.5)	11 (12.2)	**0.27 (0.10–0.72)**	0.50 (0.24–1.03)
Family size	Less than 5	13.1 (1.4)	47 (18.2)	Reference	
	At least 5	13.1 (1.5)	31 (21.3)	1.36 (0.79–2.36)	
Living conditions	With parents	13.1 (1.4)	63 (19.0)	Reference	
	With relatives	13.2 (1.5)	15 (21.1)	1.18 (0.57–2.44)	
Vegetables consumed in last 3 days	Less than 4 times	13.1 (1.4)	69 (19.4)	1.09 (0.49–2.44)	
	At least 4 times	13.2 (1.7)	9 (19.1)	Reference	
Fruit consumed in last 3 days	Less than 3 times	13.1 (14.5)	74 (19.4)	1.69 (0.49–5.75)	
	At least 3 times	12.7 (1.4)	4 (30.7)	Reference	
Daily meal frequency	Less than 3 times	12.9 (1.4)	18 (22.2)	0.84 (0.45–1.56)	
	At least 3 times	13.2 (1.4)	60 (18.7)	Reference	
Soap hand washing	Never or sometimes	13.2 (1.4)	61 (18.6)	1.39 (0.69–2.49)	
	Yes, always	13.0 (1.5)	17 (22.9)	Reference	
School location	Nalao	12.8 (1.1)	6 (15.0)	Reference	
	Sakar	13.4 (1.3)	5 (12.5)	0.75 (0.18–2.99)	
	Xokxay	13.5(1.5)	6 (13.3)	0.80 (0.20–3.13)	
	Lak 52	13.4 (1.5)	15 (19.5)	2.58 (0.70 − 9.41)	
	Pholxay	12.7 (1.4)	17 (28.8)	2.69 (0.86–8.38)	
	Sensaard	13.0 (1.4)	9 (22.5)	1.40 (0.36–5.35)	
	Lak 67	13.1 (1.6)	12 (20.0)	1.95 (0.55–6.83)	
	Pholhong	13.2 (1.4)	8 (19.5)	1.35 (0.37–4.97)	

*p < 0.05; ª Adjusted for covariates with p < 0.2 (sex, HAZ, and ethnicity) using multiple logistic regression. **At least one parasite.

## Discussion

This cross-sectional study investigated nutrition and in particular anaemia among 402 school-going adolescents, aged 10 to 18 years, residing in eight communities in Pholhong district, Vientiane province, central Lao PDR. The prevalence of anaemia was 19.4%, much lower than the national level reported in the Lao Social Indicator Survey (LSIS) II in 2017, which reported that 42.6% of adolescent girls between 15 and 18 years were anaemic. Among the girls in this study, 20.9% of adolescents 15–18 years and 17.9% of adolescents 10–14 years were anaemic. These results are comparable with the 22.2% prevalence of anaemia among 10–14 year old girls reported for Bokeo in the Northern part of Lao PDR [20]. According to the WHO classification of the severity of anaemia, we found no cases of severely anaemic adolescents. Mild anaemia was found in 20.8% of adolescents aged ≤11, 16.8% of adolescents between 12% and 14%, and 31.4% of females ≥15. The severity of anaemia among adolescents was however higher than found in the study in Bokeo, where 11.7% of adolescents had mild anaemia, but where moderate anaemia was also found, in 9.9% of adolescents of both sexes (12.0% in female and 7.2% in male adolescents) [20]. On the other hand, the severity of anaemia in the studied population was lower than found in the study in four poorest rural districts of Savannakheth province, where 75.5% of adolescent had moderate anaemia of both sexes (75.6% in male and 75.4% in female adolescents) (AGSA, Savannakheth, 2019, unpublished).

The World Health Organization estimates that around two billion individuals worldwide, i.e. over 30% of the world’s population, are anaemic, highlighting the importance of anaemia as a public health issue in both developing and developed nations [21,22]. When compared to developed countries, the prevalence of anaemia among adolescents in central Lao PDR was higher than reported for teenagers in Switzerland, 14.5% in girls and 7.9% in boys [22,23]. In Spain, Sweden, and England, the prevalence of anaemia in adolescents has been reported to be around 4.0% [24,25]. On the other hand, the prevalence of anaemia among adolescent in the central Lao PDR was lower than other countries in South Asia. In India, reported rates ranged from 30% to 56% [26,27], in rural Bangladesh it was 69% [28], in Nepal, 47.7% among male adolescents and 52.3% among females [29,30], and in Pakistan, 42% [31]. However, the prevalence rate in Lao PDR was similar to those reported for adolescents (10–19 years) in southern Ethiopia (22%) [6] and those 10–14 years in eastern Ethiopia (23.6%) [32]. That the level of anaemia among adolescents in the current study was lower than the national data may be a result of an effective national policy for prevention and control of anaemia that includes distributing weekly iron-folic acid to girls in and out of school, together with the school deworming programme, implemented by the Vientiane provincial health and education authorities. The anaemia status of respondents had no significant relationship with the presence or absence of certain intestinal infections (*Escherichia coli, Ascaris lumbricoides, Taenia* species, hookworm) which were present at low rates. This result is comparable to those from studies in other settings, for example, in Africa [6,33]. A successful school deworming programme twice per year might lead to a low prevalence rate of soil-transmitted helminthiasis. However, it was observed that *Opisthorchis viverrini* was the predominant parasite, identified in stools from nearly one-third of school adolescents. Further investigation may need to focus on eating behaviour, as raw fish, a common source of that parasite, is often consumed in southern Lao PDR.

The results revealed that more female adolescents were anaemic than males. This observation agrees with a study conducted by Galinski and Young in 2018 [34] which found that women and adolescent females often have both lower iron intake and lower total dietary intake of food products because of their dietary restraint, which directly inﬂuences their nutritional status including iron status [35]. Moreover, adolescent females are particularly susceptible to iron deﬁciency because of the combination of insuﬃcient dietary iron intake and high iron losses during menstruation [36]. Menstrual blood loss can signiﬁcantly contribute to iron depletion, as it can be diﬃcult for women to provide suﬃcient iron intake to compensate for the menstrual iron losses. This is important because all female adolescents included in the study had had their menarche, which can additionally aﬀect their iron status [5,37–39]. In addition, female adolescents and young women are more susceptible to dieting practices compared to male adolescents, which results in reduced energy intake and lower consumption of certain food products [40]. However, since females in this area were supposed to be receiving iron and folate supplement, their continuing lower iron status compared with their male counterparts is a cause for concern.

Other studies showed that adolescents in early period of adolescence (10–13 years) were about five times more likely to be anaemic compared to older adolescents (17–19), however, in the results reported here, no age difference for the prevalence of anaemia could be found [6].

Other studies have shown that the prevalence of anaemia was associated with daily meal frequency; however, we did not find any such association [6,27]. This study also did not detect any association between nutrition status and anaemia, in contrast to studies conducted in Ethiopia [6]. It was observed that 14.9% of school adolescent had BAZ greater than +2; this high rate of overweight adolescents which should be further studied, because such information might be important for designing appropriate nutrition interventions to prevent future non-communicable diseases. Also the high rate of *Opisthorchis viverini* infection is of public health concern and needs special attention to prevent future cholangiocarcinoma, which has been reported in many studies in the southern parts of Lao PDR [41–43].

This study assessed the status of anaemia in both male and female adolescents with an adequately representative sample size and methods. The sample size was not designed to investigate details of differences between the sampled schools, which would have required a much larger study. However, we acknowledge limitations such as the absence of variables on the level of education of parents, family food sources, wealth index, other hygiene practices such as wearing shoes, and dietary diversity, all of which may be associated with anaemia. We also did not investigate nutrition and anaemia of adolescents who had already left school and the study was done in one province, relatively near to the capital city. Further analysis on nutrition knowledge, eating habits, and dietary diversity among young people also in these locations will give more insight to inform the planning of interventions among this population.

## Conclusion

The prevalence of mild anaemia among both male and female school-going adolescents in the study area is a public health problem. The national nutrition programme to control and manage anaemia by distributing a weekly iron supplementation for adolescent girls together with a school deworming programme appears to have been partly successful but could be strengthened. However, intermittent iron supplementation for non-pregnant women could be more widely delivered via a range of community and health systems, including schools to reach adolescent girls, and local health workers for out of schoolgirls.

## Paper context

The government of Lao PDR has identified the need to target adolescent health concerns, in particular anaemia, due to its adverse effects on the cognitive performance, physical capacity and work performance. This study illustrates that anaemia is a mild public health problem. The national nutrition programme to control and manage anaemia, by distributing weekly iron supplementation for adolescent girls together with a school deworming programme twice per year, appears to have been partly successful but could be strengthened.
